# Oncogenic Y68 frame shift mutation of *PTEN* represents a mechanism of docetaxel resistance in endometrial cancer cell lines

**DOI:** 10.1038/s41598-019-38585-9

**Published:** 2019-02-14

**Authors:** Haiyang Zhang, Song Wang, Nicholas Cacalano, He Zhu, Qiuju Liu, Michael Xie, Mitchell Kamrava, Gottfried Konecny, Shunzi Jin

**Affiliations:** 1 Department of Prosthodontics Dentistry, Hospital of Stomatology, Jilin University, Changchun, China; 2 Department of Urology, The First Hospital of Jilin University, Changchun, China; 3 Department of Radiation Oncology, Division of Experimental Radiation Oncology, University of California, Los Angeles (UCLA), Los Angeles, California USA; 4 Department of Gynecology and Obstetrics, The Second Hospital of Jilin University, Changchun, P.R. China; 5 Tumor Center, The First Hospital of Jilin University, Changchun, P.R. China; 6 MWX Systems, Los Angeles, CA USA; 7 Medicine, Hematology & Oncology, UCLA Medical Center, Santa Monica, CA USA; 8 NHC Key Laboratory of Radiobiology, Jilin University, Changchun, China

## Abstract

In this study, we aimed to identify mutations of key genes associated with docetaxel resistance in nine endometrial cancer cell lines. Endometrial cancers are associated with several critical gene mutations, including *PIK3A, PTEN*, and *KRAS*. Different gene mutations in endometrial cancer cells have varied responses to anticancer drugs and cancer therapies. The most frequently altered gene in endometrioid endometrial carcinoma tumors is *PTEN*. PTEN protein has lipid phosphatase and protein phosphatase activity, as well as other functions in the nucleus. Although the tumor-suppressive function of PTEN has mainly been attributed to its lipid phosphatase activity, a role for PTEN protein phosphatase activity in cell cycle regulation has also been suggested. Various tumor type-specific *PTEN* mutations are well documented. Here, nine endometrioid endometrial cancer cell lines with *PIK3A, PTEN*, and *KRAS* gene mutations were treated with docetaxel and radiation. One mutation with a docetaxel drug-resistant effect was a truncated form of PTEN. Among *PTEN* mutations in endometrial cancer cells, the Y68 frame shift mutation of *PTEN* constitutes a major mechanism of resistance to docetaxel treatment. The molecular mechanism involves truncation of the 403 amino acid PTEN protein at amino acid 68 by the Y68 frame shift, leading to the loss of PTEN protein phosphatase and lipid phosphatase activities.

## Introduction

Endometrial cancer is one of the most common gynecological malignancies in women worldwide^1^. Endometrial cancers are divided two types, estrogen dependent (type I) and estrogen independent (type II). Type I is the most common type of endometrial cancer. Type II cancers include clear cell carcinoma, mucinous adenocarcinoma, and papillary serous adenocarcinoma, which are less common types of endometrial adenocarcinomas.

Early stage diseases can have good outcomes through surgery, chemotherapy, radiotherapy or hormonal therapy, while advanced diseases are more likely to recur and require adjuvant chemotherapy and radiotherapy. The combination of chemotherapy and postoperative radiotherapy has been used in the treatment of advanced endometrial cancer^2–6^. However, no standard management modality is available. Adjuvant chemotherapy and radiotherapy in the “sandwich” sequence were adopted to help identify the most effective adjuvant method for patients with advanced disease^7–11^.

Type I and type II endometrial cancers contain more than 20 gene mutations. Thus, improving our understanding of the disease at the molecular level and finding more effective strategies are important^12–14^. Currently, chemotherapeutics remains the primary treatment for endometrial cancer. However, a major problem with chemotherapeutics is drug resistance. Therefore, the identification of genetic mechanisms involved in the chemotherapeutic response is critical for predicting the drug response of tumors with gene mutations. We propose that critical mutations of the tumor suppressor gene PTEN may be the major chemotherapeutic resistant factor in the treatment of patients with docetaxel-resistant endometrial cancer.

Frequent mutations in *PIK3A, PTEN, KRAS* and *FGFR2* might affect adjuvant treatment of endometrial tumors^15–18^. Radiation therapy is a key therapeutic strategy for endometrial carcinomas. However, how different gene mutations affect radiation sensitivity and drug responses remains unknown. Currently, treatment for metastatic or recurrent disease is based on the conventional chemotherapy method. Despite the different gene mutations in endometrial cancers, most clinical treatments have not taken this diversity into account^19,20^.

Gene mutations in *PTEN* lead to deregulation of the cell cycle^21^. *PTEN* suppresses the progression of the cell cycle through reduced cyclin D1 and increased p27.

Here, we aimed to investigate the roles of *PIK3A, PTEN, KRAS* and *FGFR2* gene mutations and five different mutations of PTEN in endometrioid endometrial carcinoma (EEC) cells to identify the mechanisms of docetaxel chemotherapy and radiation therapy resistance for different mutations in endometrial carcinomas. Cells were exposed to a chemotherapy drug (docetaxel), ionizing radiation (2 Gy) or a combination of both (sandwich method). Drug responses and radiosensitizing effects were evaluated using MTT assays and xCELLigence Real-Time Cell Analysis (RTCA). The effects of treatment with different doses of the chemotherapy drug (docetaxel) were evaluated following exposure to ionizing radiation (2 Gy).

We present multiple analyses of MTT assays and xCELLigence RTCA of 9 EEC cell lines treated with docetaxel chemotherapy and radiation. This integrated analysis provides the molecular parameters of different responses of endometrial carcinoma cells with various gene alterations, which may have a direct effect on treatment recommendations for patients. Our analysis also provides references for gene mutation-based clinical practice and novel treatments involving docetaxel chemotherapy and radiation.

## Materials and Methods

### Cell lines and reagents

The effects of docetaxel on malignant cell growth were studied in a panel of 9 established human endometrial cancer cell lines. The individuality of each cell line was confirmed by mitochondrial DNA sequencing immediately after receipt from the collaborating research laboratory. Cell lines were passaged for less than 6 months after authentication and SPAC-1-L cell line was confirmed by PCR and sequencing experiments. Ishikawa cells were obtained from the European Collection of Animal Cell Cultures. The established human endometrial carcinoma cell line HEC155 was obtained from the Japanese Health Science Research Resources Bank. The cell line SPAC-1-L was provided by the laboratory of Dr. Y. Hirai from the Department of Gynecology, Cancer Institute Hospital (Tokyo, Japan). Dr. A. Santin provided ARK1 (USPC1) and ARK2 (USPC2) cells from the Division of Gynecologic Oncology at the University of Arkansas (Little Rock, AR). The cell lines were cultured in Modified Eagle’s Medium (MEM) supplemented with 10% heat-inactivated FBS, 2 mmol/L glutamine and Antibiotic-Antimycotic Solution (Mediatech, Inc. Manassas, VA)^21^.

### Cell viability assays following radiation and docetaxel

Cells were plated in 96-well plates at a density of 2,000 cells per well, and untreated control cells were optimized to 85 to 95% confluence at the endpoint of the experiment. After 24 hours, cells were treated with radiation doses (2 Gy) and different concentrations (1 ng/mL to 4,000 ng/mL, Table 1) of docetaxel. ARK1 cells were treated with drug concentrations of 1, 2, 3, 4 and 5 ng/mL; ISHIKAWA cells were treated with drug concentrations of 1, 2, 4, 6, and 8 ng/mL; ARK2 cells were treated with drug concentrations of 2, 4, 6, 8 and 10 ng/mL; HEC155 cells were treated with drug concentrations of 0.5, 1, 5, 10 and 20 ng/mL; and SPAC-1-L cells were treated with drug concentrations of 10, 20, 40, 80 and 100 ng/mL. After two and five days of treatment, cell viability was assessed by a MTT cell proliferation assay (Promega). Cell lines that failed to achieve the IC_50_ for a given drug were nominally assigned the highest concentration screened (i.e., 100 ng/mL). Experiments were performed in triplicate for each cell line. The association between a cell line and the drug response was determined using a two-tailed *P* value less than 0.05, which was considered statistically significant.

**Table 1:** Drug concentrations of docetaxel.

Table 1	Control	Drug D (ng/ml)					IR (2 Gy)	Drug D + IR (2 Gy)				
Cells	C0	C1	C2	C3	C4	C5	C0 + 2 Gy	C1 + 2 Gy	C2 + 2 Gy	C3 + 2 Gy	C4 + 2 Gy	C5 + 2 Gy
ARK1	0	1	2	3	4	5	0	1	2	3	4	5
ARK2	0	2	4	6	8	10	0	2	4	6	8	10
ECC-1	0	5	10	20	30	40	0	5	10	20	30	40
HEC155	0	0.5	1	5	10	20	0	0.5	1	5	10	20
HEC-1-A	0	0.5	1	5	10	20	0	0.5	1	5	10	20
ISHIKAWA	0	1	2	4	6	8	0	1	2	4	6	8
MFE319	0	1	2	3	4	5	0	1	2	3	4	5
SNG-II	0	1	2	3	4	5	0	1	2	3	4	5
SPAC-1-L	0	10	20	40	80	100	0	10	20	40	80	100

### Real-time cell-based analysis by an xCELLigence System

An xCELLigence Real-Time Cell Analyzer System (ACEA Biosciences San Diego, CA) was used according to the manufacturer’s instructions. Briefly, the working mechanism of the system can be summarized as follows. The xCELLigence System allows continuous measurement and quantification of cell adhesion and proliferation. This system uses 96-well E-plates, and these disposable plates are suitable for single use. On the bottom of the wells, the plates have a base containing gold microelectrodes. There is 70% electrode coverage on the bottom of the wells. The electrical impedance of these sensor electrodes is recorded to monitor the changes in the cells. Changes in electrical impedance are presented with a parameter called the “cell index (CI)”. When there are no cells in the wells, electrode impedance and the CI are zero. After seeding cells, the CI will increase. The increase in the CI correlates with the increase in attached cell numbers. When more cells are attached to the surface of the E-Plate, the CI increases. Additionally, instead of cell numbers, cell viability and the strength of cell adhesion can cause changes in the CI^22,23^.

A cell proliferation experiment was performed using an xCELLigence System. When EEC cells reached 80% confluence in a 6-well plate, the cells were washed with PBS. Then, the cells were treated with 0.05% trypsin/EDTA. After 2 min, 5 mL complete medium was added to the wells. The cell resuspension was centrifuged at 400 × g for 5 min. The pellet was resuspended in 5 mL media, and the cells were counted by using a hemocytometer. A total of 5,000 cells per well were seeded in an E-Plate, and the total volume of the wells was adjusted to 100 µL with media. The E-Plate was incubated overnight in a cell culture incubator. Before measuring the cells in the E-Plate, a standard background was measured by adding 50 μL complete medium at 37 °C to the wells. EEC cells were monitored every 15 min for 144 hours. Data are presented as a normalized CI.

## Results

### Multiple gene mutations in EEC cell lines

#### Drug concentration-dependent responses of EEC cells to docetaxel

PTEN gene mutations are common in EEC cell lines. We investigated the responses of nine EEC cell line panels to docetaxel chemotherapy and radiation to determine docetaxel drug resistance in association with *PIK3A, PTEN* and *KRAS* mutations. Four cell lines (ARK1, ARK2, HEC155, and SPAC1L) were obtained from patients with type II uterine serous papillary endometrial carcinoma. The MFE319 cell line originates from an endometrial adenosquamous carcinoma. The remaining cell lines are derived from type I EECs^24^. Among these cell lines, five out of nine (56%) EEC cell lines harbor *PTEN* gene mutations in different positions (ECC-1/V317 frame shift, ISHIKAWA/V317 frame shift, MFE319/V317 frame shift, SNG-II/E288 frame shift and SPAC-1-L/ E288 frame shift/Y68 frame shift). We irradiated each of the 9 EEC cell lines with 0, 1, 2, 4, 6, 8, 11, or 15 Gy of X-rays and measured by MTT. The results showed the irradiation dose of 2 Gy not only partially reduced survived but also reserved a fraction room for docetaxel chemotherapeutics. Therefore the irradiation 2 Gy is an appropriate dose for the studying combination of irradiation and chemotherapeutics of docetaxel to EEC cells. We exposed nine EEC cell lines to different concentrations of the chemotherapy drug docetaxel, radiation (2 Gy) and both treatments for two or five days (mean of at least 3 independent experiments performed in triplicate), followed by MTT assays.

The effective dose range for drug docetaxel (IC_10_-IC_90_) was identified using a wide range of drug concentrations (0.5–100 ng/ml). Docetaxel inhibited the proliferation of all endometrial cancer cell lines investigated in a concentration-dependent manner. However, the IC_50_ values significantly varied between individual cell lines with up to a 50-fold difference in the IC_50_ values and ranged between 1.95 in ARK1 endometrial cells with a *PI3K* gene mutation and 105.82 in SAPC-1-L cells that harbor a *PTEN* mutation (E288 frame shift /Y68 frame shift) (Table 2). ECC-1 cells with a *PTEN* mutation (V317 frame shift, exon 8) were less sensitive to docetaxel with a mean IC50 of 18.2 ng/ml, while ECC1 cells were sensitive to 2 Gy radiation with a relative viability ratio of 0.29. HEC155 cells were less sensitive to docetaxel with an IC_50_ value of 19.7 ng/ml, but these cells were sensitive to radiation with a relative viability ratio of 0.48 with a 2 Gy irradiation dose.

**Table 2:** Mutations of EC cell lines and IC_50_

	P13K			PTEN			KRAS			IC50	Viability	Viability
	Nucleotide change and position in Ref mRNA	Amino acid change	Exon	Nucleotide change and position in Ref mRNA	Amino acid change	Exon	Nucleotide change and position in Ref mRNA	Amino acid change	Exon	Drug D (ng/ml) (2 Days) (±SE)	IR (2 Gy) (2 Days) (±SE)	IR (2 Gy) (5 Days) (±SE)
ARK1	G1624A	E542K	X10							1.95 ± 0.47	0.80 ± 0.10	0.54 ± 0.17
ARK2										5.26 ± 0.41	0.70 ± 0.08	0.29 ± 0.11
ECC-1				Tact950−	V317 frame shift	X8				18.21 ± 1.99	0.93 ± 0.12	0.29 ± 0.15
HEC155										19.65 ± 1.23	0.94 ± 0.06	0.48 ± 0.15
HEC-1-A	G3145C	G1049R	X21				G35T	G12V	Exon2	17.26 ± 1.66	0.96 ± 0.05	0.28 ± 0.13
ISHIKAWA				TACT950−	V317 frame shift	X8				7.36 ± 0.58	0.65 ± 0.10	0.48 ± 0.09
MFE319				TACT950−	V317 frame shift	X8				5.28 ± 0.28	0.98 ± 0.10	0.75 ± 0.06
SNG-II				A863−	E288 frame shift	X8	G35T	G12V	Exon2	2.63 ± 0.30	0.71 ± 0.07	0.38 ± 0.04
SPAC-1-L				A863−	E288 frame shift	X8				105.8 ± 5.68	0.94 ± 0.21	0.57 ± 0.26
				−202GATA	Y68 frame shift	X3						

^*^IC_50_ values were measured in EEC cells that were treated with docetaxel for two days, and cell viabilities were measured two and five days after irradiation with a 2 Gy dose.

#### Effects of docetaxel on endometrial cancer cells with *PTEN* mutations

**Figure 1:**
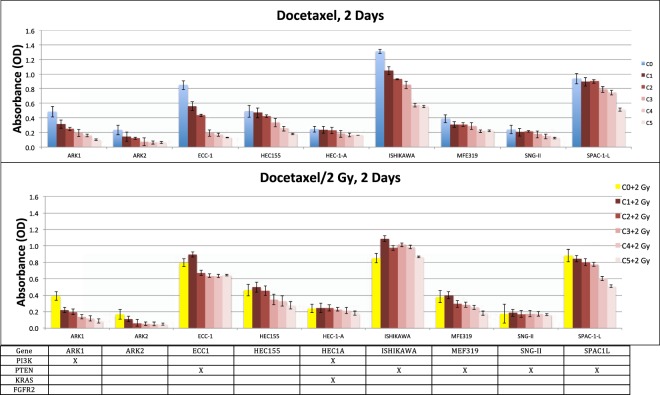
Drug concentration-dependent response of EEC cells to docetaxel and combination with radiation (2 Gy) in the sandwich method associated with mutation patterns. Viabilities of 9 EEC cell lines treated for two days with serial dilutions of docetaxel relative to those of untreated cells were determined by MTT assays (mean of at least 2 independent experiments in triplicate). Gene mutations of each cell line are shown below the graph where columns represent individual cell lines, rows represent genes, and x indicates the presence of a mutation.

Exposure of ECC-1, ISHIKAWA, MFE319, SNG-II and SPAC-1-L human endometrial cancer cells with *PTEN* mutations to docetaxel resulted in a dose-dependent reduction in cell viability. MFE319, ISHIKAWA and ECC-1 cells with a *PTEN* mutation (V317 frame shift, exon 8) responded to docetaxel with low IC_50_ values of 5.28 and 7.36 to higher values of 18.21 ng/ml. ISHIKAWA and MEF319 cells were sensitive to docetaxel in a dose-dependent fashion from a low drug concentration of 1 ng/ml (Fig. 1), with relative viability ratios of 0.92 and 0.90. However, ECC-1 cells were not very sensitive to docetaxel, showing a dose-dependent reduction and higher drug IC_50_ value of 18.21 ng/ml, with a relative viability ratio of 0.89 with a dose of 5 ng/ml. SNG-II cells with a *PTEN* mutation (E288 frame shift) were sensitive to docetaxel with a dose-dependent reduction from a low drug concentration of 1 ng/ml, with a drug IC_50_ value of 2.63 ng/ml. Additionally, SAPC-1-L cells that harbor a *PTEN* mutation (E288 frame shift/Y68 frame shift) were resistant to docetaxel, exhibiting a dose-dependent reduction with a high drug IC_50_ value of 105.8 ng/ml, which suggests that the Y68 frame shift mutation of *PTEN* may be associated with docetaxel resistance in endometrial cancer cells.

#### Combinations of docetaxel chemotherapeutics and radiation therapy

**Figure 2:**
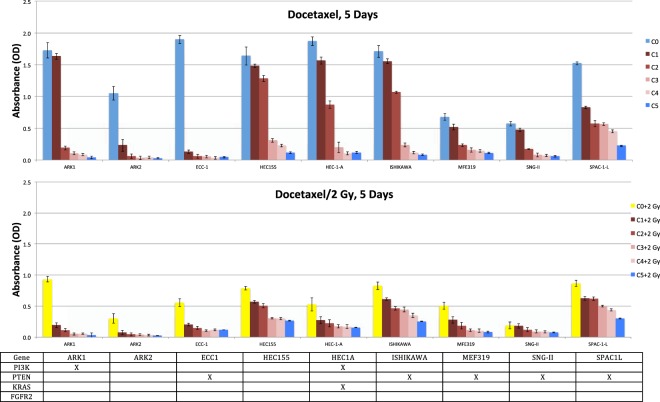
Drug concentration-dependent response of EEC cells to docetaxel and combination with radiation (2 Gy) in the sandwich method associated with mutation patterns. Viabilities of 9 EEC cell lines treated for five days with serial dilutions of docetaxel relative to those of untreated cells were determined by MTT assays (mean of at least 2 independent experiments in triplicate). Gene mutations of each cell line are shown below the graph where columns represent individual cell lines, rows represent genes, and x indicates the presence of a mutation.

Because SPAC-1-L cells were highly resistant to docetaxel, we sought other methods (radiation) to more effectively kill these cells. The survival rates of ARK1, ARK2, ECC-1, HEC155, HEC-1-A, ISHIKAWA, SNG-II and SPAC-1-L cells ranged from 0.65 to 0.96 with a 2 Gy dose for two days and from 0.28 to 0.57 with a 2 Gy dose for five days. MFE319 cells were resistant to radiation (2 Gy), with survival rates of 0.98 for two days and 0.75 for five days (Table 2). However, little is known regarding the potential synergistic effect of docetaxel plus radiation on killing endometrial cancer cells with gene mutations. Here, we investigated whether the combination of docetaxel and ionizing radiation enhanced the destruction of endometrial cancer cells more than each modality individually. The docetaxel concentrations used for the experiments ranged from 0.5 ng/ml to 100 ng/ml. Synergistic and additive interactions were observed in two endometrial cancer cells, ARK1 and ARK2, with a dose-dependent reduction for docetaxel plus irradiation (2 Gy) for two days (Fig. 1) and five days (Fig. 2).

#### Cell cycle with the Y68 frame shift mutation of PTEN, docetaxel chemotherapeutics and radiation therapy

**Figure 3:**
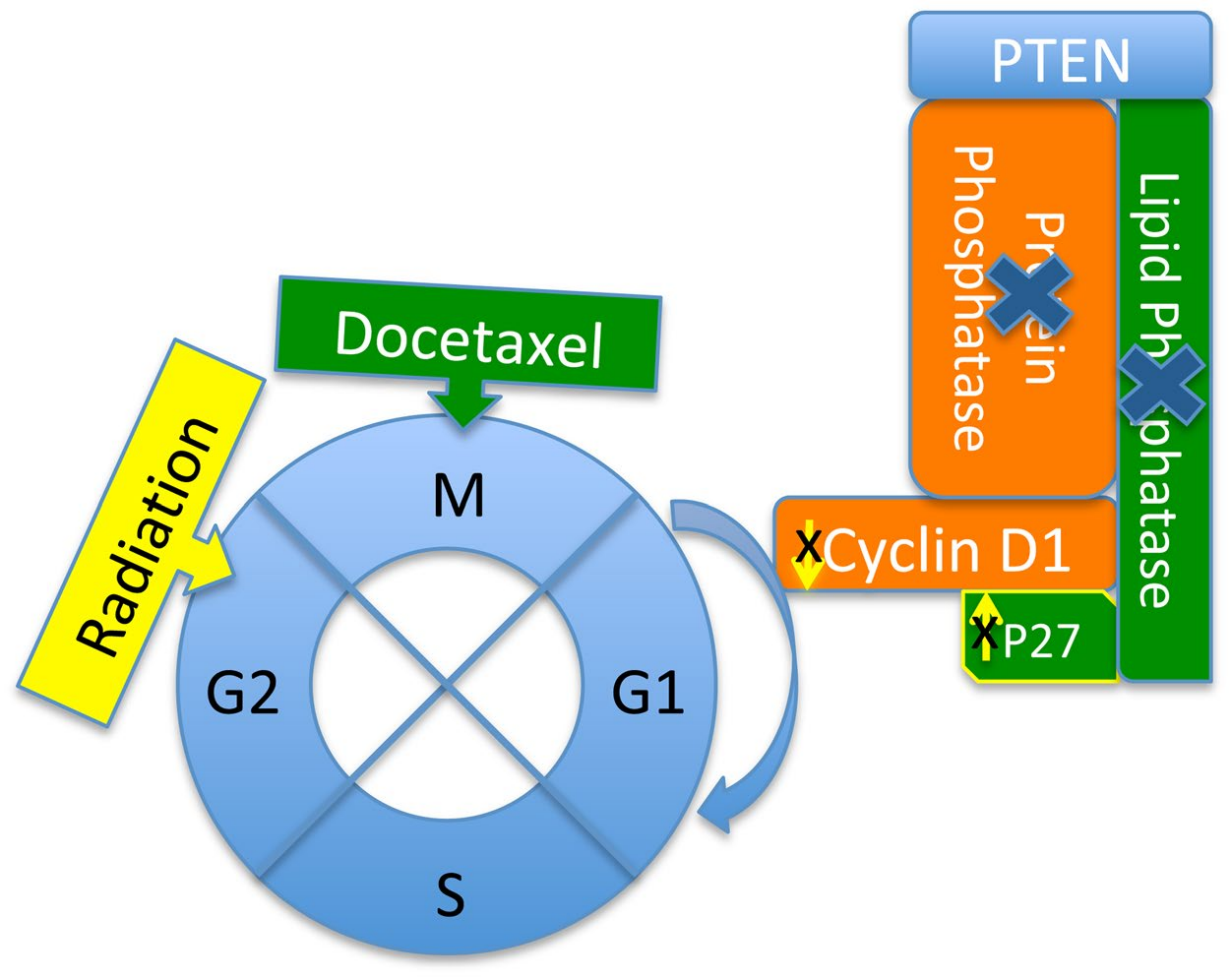
Y68 frame shift mutation results in the loss of PTEN protein phosphatase and lipid phosphatase activities, which block cell cycle progression by downregulating the positive cell cycle regulator cyclin D1 and by upregulating the negative cell cycle regulator p27. Loss of control of cyclin D1 and p27 activities by PTEN alters the effectiveness of docetaxel chemotherapeutics and radiation therapy.

Molecular mechanism of resistance to docetaxel in the SPAC-1-L cell line with the Y68 frame shift mutation of *PTEN*: PTEN is a dual-specificity phosphatase with protein phosphatase and lipid phosphatase activities, which demonstrate that PTEN blocks cell cycle progression by downregulating the positive cell cycle regulator cyclin D1 through its protein phosphatase activity and upregulating the negative cell cycle regulator p27 through its lipid phosphatase activity^9,25–27^. Due to the loss of protein phosphatase and lipid phosphatase activities, PTEN loses the ability to regulate cyclin D1 and p27 and then loses control of the cell cycle through regulation of G1. Docetaxel-induced microtubule stabilization arrests cells in the G2/M phase of the cell cycle and leads to apoptotic death of cancer cells (Fig. 3). Docetaxel’s mechanism of action against tumor cells is alteration of microtubule dynamics, which causes cell cycle arrest during mitosis. Docetaxel binds to microtubules with a higher affinity than does paclitaxel and over a broader range of cell cycle activities.

Due to the loss of the core motif (P loop, HCKAGKGR, residues 123–130) and C2 tensin-type domain (residues 186–351), the Y68 frame shift leads to the loss of the protein phosphatase and lipid phosphatase activities of *PTEN* and is predicted to be much more resistant to docetaxel. Four cell lines, ECC-1, ISHIKAWA, MFE319 and SNG-II, have the V317 frame shift and E288 frame shift of *PTEN* C2 domain mutations and are predicted to be less resistant to docetaxel. We tested the responses of nine endometrial cancer cell lines to the chemotherapy drug docetaxel. Among these cell lines, five out of nine (56%) EC cell lines harbored *PTEN* gene mutations in different amino acid change positions (ECC-1/V317 frame shift). The docetaxel IC_50_ values of the ECC-1, ISHIKAWA, MFE319, and SNG-II cell lines with *PTEN* mutations were 18.21 ng/ml, 7.36 ng/ml, 5.28 ng/ml and 2.63 ng/ml, respectively. These IC_50_ values were similar to those of cell lines with other gene mutations (ARK1/1.95 ng/ml, ARK2/5.26 ng/ml, HEC155/19.65 ng/ml and HEC-1-A/17.26 ng/ml). The docetaxel IC_50_ value of SPAC-1-L with the Y68 frame shift mutation of *PTEN* was 105.8 ng/ml, which was five times greater than the IC_50_ values of cell lines with other *PTEN* mutations and other gene mutations.

In this study, we found an association between the Y68 frame shift and increased resistance to docetaxel. Our preclinical *in vitro* findings demonstrate that loss of PTEN expression with the Y68 frame shift mutation predicts a lack of benefit from docetaxel in patients during endometrial cancer chemotherapy. Therefore, other chemotherapy drugs should be considered instead of docetaxel. This novel potent biomarker might lead to further refinement of therapy for patients with endometrial cancer with a Y68 frame shift mutation of *PTEN*, facilitating better outcomes, a better toxicity profile, and more effective health care delivery. Due to the relevance of the Y68 frame shift mutation and docetaxel resistance in endometrial cancer, this mutation is a predictive marker worth further preclinical and clinical evaluation.

## Discussion

Since cancer is one of the leading causes of death worldwide, finding better treatments is an urgent need. Although remarkable progress has been made towards understanding the molecular and cellular mechanisms of cancer development and treatment in recent years, the clinical management of cancer remains a challenge. Currently, chemotherapeutics and radiation therapy remain the predominant options for cancer therapy. However, resistance to chemotherapy in cancer is common. Therefore, the identification of gene mutations involved in the chemotherapeutic response is critical for predicting tumor responses and treating patients with drug-resistant cancer with different gene mutations. In this study, we sought to identify genetic mutations associated with resistance to facilitate effective combination therapy.

Therapeutic strategies could be improved according to appropriate biomarkers and patient-specific mutation profiles to achieve more benefits from combination therapies. The PI3K pathway is frequently activated in most EECs^18,28^. Mutations of the *RAS* gene are also very common, accounting for more than 20% of mutations in human tumors^29^. Moreover, the fibroblast growth factor receptor (FGFR) pathway is involved in the pathogenesis and progression of endometrial cancer^30–32^. These gene mutations hold great promise for more effective treatment strategies for this disease.

Most endometrial carcinomas are diagnosed at an early stage. Nevertheless, 15–20% of these carcinomas recur with limited effects of systemic therapies in metastatic disease. Recent progress in the identification of genetic abnormalities in endometrial cancer has spurred the development of novel strategies for the treatment of patients with endometrial cancer. Novel strategies involving therapies with greater efficacy and reduced toxicity are needed. Currently, mutation-based treatments are not available in the clinic; thus, more systematic approaches to the integration of biomarkers in clinical trials of therapeutic agents are needed for clinical implementation.

To date, no studies have investigated the responses of endometrial cancer cells with different gene mutations to docetaxel chemotherapy. Here, we show a concentration-dependent reduction in the viability of nine endometrial cancer cell lines in response to docetaxel. However, the response significantly varied among individual cell lines, with differences of up to 40 times in IC_50_ values. Surprisingly, significantly higher IC_50_ values were associated with a Y68 frame shift mutation of PTEN in SPAC-1-L cells. The role of the Y68 frame shift mutation of PTEN in endometrial cancer has not yet been studied. However, our preclinical findings suggest that the PTEN Y68 frame shift mutation is associated with resistance to docetaxel in endometrial cancer.

PTEN is an essential tumor suppressor gene that antagonizes the PI3K/Akt/mTOR antiapoptotic pathway and is often abnormal in endometrial cancers. Impairment of this tumor suppressor pathway potentially becomes a causal factor for the development of malignancies. Activation of the PI3K/Akt/mTOR pathway is implicated in the pathogenesis of malignancies and the development of resistance to anticancer therapies^33^.

ARK1 cells with a *PI3K* gene mutation are sensitive to docetaxel with an IC_50_ of 1.95 ng/ml, while ECC-1 cells with a *PTEN* mutation are nine times more resistant to docetaxel with an IC_50_ of 18.21 ng/ml (Table 2).

**Figure 4:**
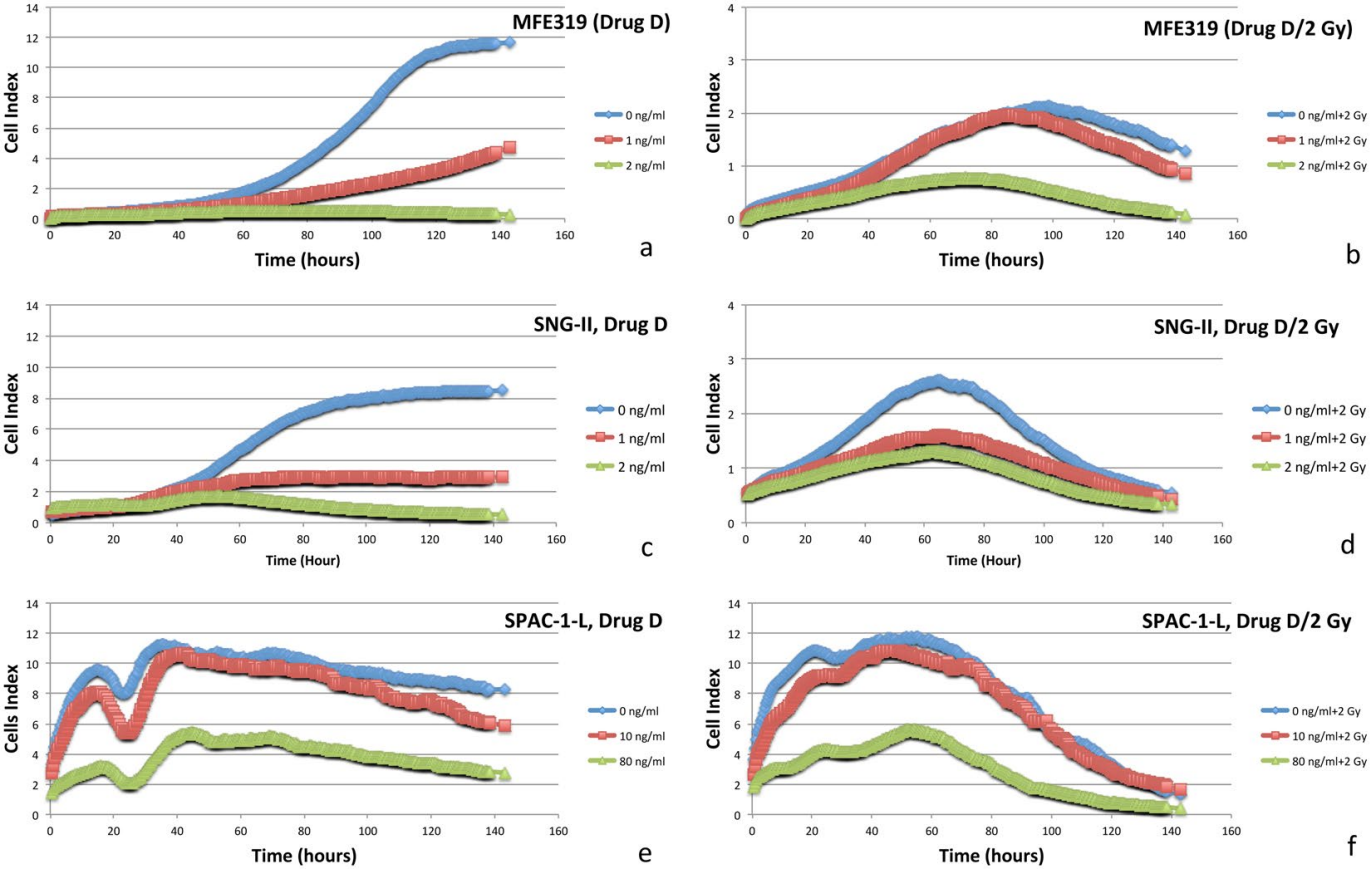
MFE319 (V317 frame shift) cells: real-time response curves of docetaxel and radiation (**a,b**); SNG-II (E288 frame shift) cells: real-time response curves of docetaxel and radiation (**c,d**); SPAC-1-L (E288 frame shift/Y68 frame shift) cells: real-time response curves of docetaxel and radiation (**e,f**). Curves are data from the xCELLigence Real-Time Cell Analyzer System.

For MFE319 (V317 frame shift), SNG-II (E288 frame shift) and SPAC-1-L (E288 frame shift/Y68 frame shift) cells with a *PTEN* mutation, the radiation effect started after two days of irradiation (2 Gy) based on the real-time response curves of radiation. When docetaxel concentrations were higher than 1 ng/ml, the drug achieved a greater therapeutic effect than did the combination of drug and radiation (Fig. 4). Compared to the SNG-II (E288 frame shift) cell line, the SPAC-1-L (E288 frame shift/Y68 frame shift) cell line was 40 times more resistant to docetaxel after adding the Y68 frame shift, which implies that the Y68 frame shift mutation of *PTEN* plays a crucial role in docetaxel resistance.

The main goal of radiation therapy is to kill cancer cells by destroying their multiplication (cell division) potential. Resistance to chemotherapy and radiation therapy is a major problem facing current cancer research. In the current study, we evaluated the therapeutic effect of docetaxel with or without the addition of radiation (X-ray, 2 Gy) in nine endometrial cancer cell lines.

**Figure 5:**
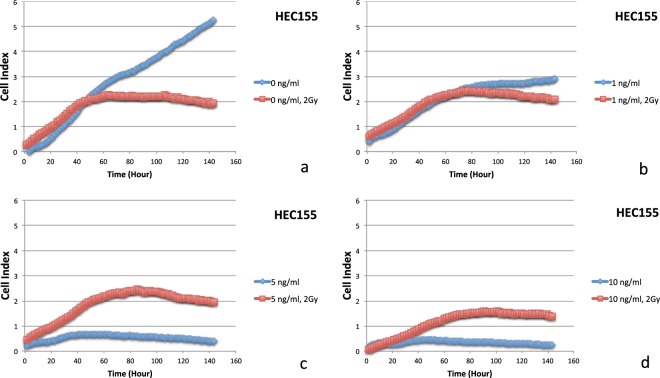
Data of the xCELLigence Real-Time Cell Analyzer System: HEC155 cells: real-time response curves of docetaxel ((**a**) 0 ng/ml, (**b**) 1 ng/ml, (**c**) 5 ng/ml and (**d**) 10 ng/ml) and radiation (2 Gy).

**Figure 6:**
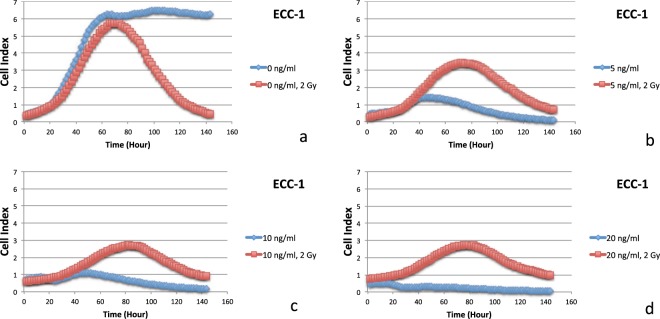
Data of the xCELLigence Real-Time Cell Analyzer System: ECC-1 cells: real-time response curves of docetaxel ((**a**) 0 ng/ml, (**b**) 5 ng/ml, (**c**) 10 ng/ml and (**d**) 20 ng/ml) and radiation (2 Gy).

**Figure 7:**
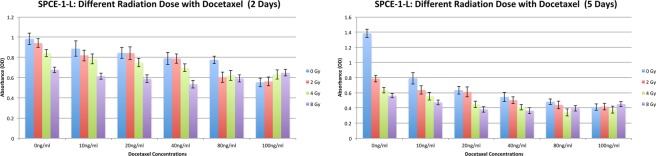
SPAC-1-L cells: Different radiation doses (0 Gy, 2 Gy, 4 Gy and 8 Gy) with different doses (0 ng/ml, 10 ng/ml, 20 ng/ml, 40 ng/ml, 80 ng/ml and 100 ng/ml) of docetaxel.

An antagonistic interaction was observed for docetaxel plus radiation (2 Gy) in ECC-1, HEC155, HEC-1-A, ISHIKAWA, MFE319, SNG-II and SPAC-1-L cells. In Fig. 1 (two days), Fig. 2 (five days), Figs 5 and 6, HEC155 and ECC-1 cells exhibited antagonistic interactions at a concentration of 5 ng/ml. As shown in Fig. 1 (two days), Fig. 2 (five days) and Fig. 7, SPAC-1-L cells exhibited antagonistic interactions at concentrations of 80 ng/ml~100 ng/ml at 2 Gy. Radiation blocks the cell cycle at the G2 phase, while docetaxel blocks the cell cycle at M phase. The radiation interferes the cytotoxic effects of docetaxel on the mitotic arrest. Results showed that the doses of antagonistic interactions are depending on resistances to docetaxel of different EEC cell lines. These conditions resulted in a cell cycle-dependent antagonistic interaction that affected the antitumor activity of docetaxel^34–37^. Given the known molecular mutations, we hypothesized that EEC cell lines with activating the Y68 frame shift mutation of *PTEN* are resistant to irradiation therapy compared to EEC cell lines with wild-type *PTEN*.

HEC-1-A cells were less sensitive to docetaxel with an IC_50_ value of 17.3 ng/ml. Since HEC-1-A cells were with *PI3K* and *KRAS* mutations and wild type *PTEN*, they were more sensitive to radiation with a relative viability ratio of 0.28 with a 2 Gy irradiation dose. SPAC-1-L is mutated with the Y68 frame shift mutation of *PTEN*, and then it is more resistant to radiation. This result supports hypothesis that SPAC-1-L is mutated with the Y68 frame shift mutation of *PTEN* induces docetaxel resistance in endometrial carcinoma cells.

Based on data from the xCELLigence Real-Time Cell Analyzer System, an antagonistic interaction was observed for docetaxel plus irradiation (2 Gy) in a dose-dependent manner with drug concentrations of 0, 1, 5, and 10 ng/ml in the HEC155 cell line (Fig. 5) and concentrations of 0, 5, 10, and 20 ng/ml in ECC-1 cells with a PTEN mutation (Fig. 6) for five days. Radiation can also cause cell cycle perturbations, such as G1 or G2-M phase delay. Based on data from the xCELLigence Real-Time Cell Analyzer following radiation exposure (X-ray, 2 Gy), both docetaxel and radiation (2 Gy) reduced cell viability (Figs 5 and 6). Interestingly, far fewer cells underwent apoptosis among the cells exposed to the combination of docetaxel (more than 5 ng/ml) and radiation (2 Gy). This result indicates that radiation might interfere with the ability of docetaxel to induce mitotic arrest and apoptotic cell death. Moreover, this result may explain why the combination of radiation and docetaxel results in a cell cycle-dependent reduction.

Our results showed that the Y68 frame shift mutation of the *PTEN* gene led to docetaxel drug resistance in chemotherapy of endometrial cancer cells by destroying the protein phosphatase and lipid phosphatase activities of PTEN. Compared with treatment with docetaxel alone, the combination of docetaxel and X-ray (2 Gy) radiation resulted in a significant decrease in overall cytotoxicity for most endometrial cancer cells. The radiation sensitizing effects of docetaxel on endometrial cancer cells were observed at very low drug levels, far below the levels required for cytotoxic effects. Our results demonstrated that the addition of X-ray radiation interfered with the cytotoxic effects of docetaxel and might provide further proof for the cell cycle-dependent interaction.
